# RNA in axons, dendrites, synapses and beyond

**DOI:** 10.3389/fnmol.2024.1397378

**Published:** 2024-09-18

**Authors:** Richard Taylor, Nikolas Nikolaou

**Affiliations:** ^1^Centre for Developmental Neurobiology, Institute of Psychiatry, Psychology and Neuroscience, King’s College London, London, United Kingdom; ^2^Department of Life Sciences, University of Bath, Bath, United Kingdom

**Keywords:** messenger RNA (mRNA), local mRNA translation, cleavage and polyadenylation, stability and degradation, intron retaining RNA (IR RNA), microRNA (miRNA), long non coding RNA (lncRNA), circular RNA (circRNA)

## Abstract

In neurons, a diverse range of coding and non-coding RNAs localize to axons, dendrites, and synapses, where they facilitate rapid responses to local needs, such as axon and dendrite extension and branching, synapse formation, and synaptic plasticity. Here, we review the extent of our current understanding of RNA subclass diversity in these functionally demanding subcellular compartments. We discuss the similarities and differences identified between axonal, dendritic and synaptic local transcriptomes, and discuss the reported and hypothesized fates and functions of localized RNAs. Furthermore, we outline the RNA composition of exosomes that bud off from neurites, and their implications for the biology of neighboring cells. Finally, we highlight recent advances in third-generation sequencing technologies that will likely provide transformative insights into splice isoform and RNA modification diversity in local transcriptomes.

## Introduction

Neurons are highly polarized cells with often sophisticated morphologies, resulting in their axons, dendrites, and synapses (collectively termed neurites) being situated several millimeters from the soma. In some cases axons extend beyond a meter, and dendrites over a centimeter (Holt et al., 2019). As functionally and metabolically demanding cell compartments (Harris et al., 2012; Faria-Pereira and Morais, 2022; Yang et al., 2023), neurites require highly efficient protein production and cycling for their development and maintenance. This demand calls for elaborate mechanisms beyond centralized production in the soma and subsequent delivery to neurites (Hanus and Schuman, 2013). Exclusively somatic protein synthesis would delay any changes to local proteomes required for dynamic responses to locally received stimuli (Fonkeu et al., 2019). Furthermore, the short half-life of many neurite-localized proteins indicates they would not survive a journey centimeters in length, or not last long following their arrival (Piper and Holt, 2004; Sun and Schuman, 2022).

However, over the last few decades, extensive decentralization of these processes has been uncovered (Holt et al., 2019; Sun and Schuman, 2023). The delivery of ribonucleic acid (RNA) molecules to the far-flung limits of neurons enables agile, responsive, on-site production of proteins exactly when they are required. Early studies utilizing *in situ* hybridisation identified numerous messenger RNAs (mRNAs) localized to neurites. More recently, a plethora of high-throughput sequencing studies have more thoroughly characterized local transcriptomes, providing detailed global insight into the different types of RNAs enriched in neurites, revealing those that are common, as well as those specifically enriched in either axons, dendrites, or synapses. Such studies have utilized various mammalian and non-mammalian sample types, including compartmentalized culture of embryonic stem cell (ESC)- and induced pluripotent stem cell (iPSC)-derived neurons, embryonic and adult primary neurons, dorsal root ganglia (DRG) explants, or dissection of neuropil (axon- and dendrite-enriched tissue).

Whilst most studies characterizing axonal, dendritic, and synaptic transcriptomes thus far have focused on mRNA expression, this accounts for up to only 5% of total RNA in a cell, with the rest being non-coding RNAs (ncRNAs)−predominantly ribosomal RNA (rRNA) and transfer RNA (tRNA) (Wu et al., 2014; Deng et al., 2022). However, the proportions of each type of RNA specifically within axons, dendrites, and synapses is unknown. Indeed, more recently an increasing number of studies have turned their focus towards elucidating diversity amongst local ncRNAs.

**FIGURE 1:**
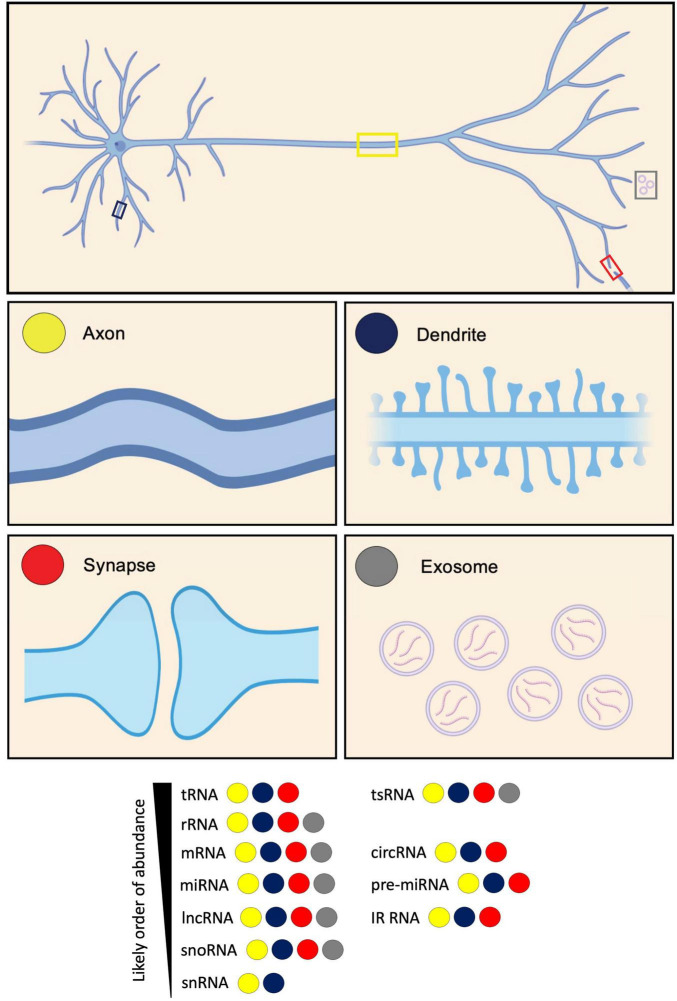
Diversity of RNA types present in neurites and exosomes. RNA types categorized based on their identification in axons, dendrites, synapses, and exosomes. These RNA types include: transfer RNA (tRNA); tRNA-derived small RNA (tsRNA); ribosomal RNA (rRNA); messenger RNA (mRNA); circular RNA (circRNA); microRNA (miRNA); pre-microRNA (pre-miRNA); long non-coding RNA (lncRNA); intron-retained RNA (IR-RNA); small nucleolar RNA (snoRNA); and small nuclear RNA (snRNA). RNA types are listed according to their likely order of abundance.

In this review, we highlight the various classes of RNAs that localize to axons, dendrites and synapses, as well as exosomes, which enable the transfer of RNAs to neighbouring cells when secreted (Figure 1). We summarize the key datasets characterizing the classes found within each subcellular compartment across different sample types. We subsequently compare the transcriptomes for the different subcellular compartments. We go on to discuss the fates and functions of the different identified RNA classes, and their implications for the development and maintenance of each respective compartment. Finally, we outline recent advances in third-generation sequencing technologies, that hold the power to revolutionize our understanding of splice isoform diversity and RNA modifications in local transcriptomes.

## Messenger RNAs (mRNAs)

### mRNA diversity in axons, dendrites, and synapses

The most extensively studied RNAs in neurites are those that encode proteins. mRNAs were first identified within dendrites by *in situ* hybridization (Davis et al., 1987; Garner et al., 1988), and later in axons [reviewed in Steward (1997); Figure 1]. Before these findings, it was assumed that all neurite-localized proteins were trafficked from the soma (Alvarez et al., 2000). Our first insight into the notion of local translation was the observation of polysomes sitting immediately beneath post-synaptic sites within dendrites (Steward and Levy, 1982; Eberwine et al., 2001). Later, mRNAs were shown to associate with polysomes and undergo translation, underpinning plasticity (Holt and Schuman, 2013). More recently, monosomes were discovered to form the dominant ribosomal population within neurites (Biever et al., 2020).

Below, we outline the main findings from key studies characterizing the transcriptomes of axons, dendrites, and synapses. We compare datasets on a compartment-specific basis, before going on to compare axonal versus dendritic versus synaptic transcriptomes.

While early studies identified mRNAs for a small number of genes in neurites by *in situ* hybridisation, more recent studies have utilized high-throughput bulk RNA-Seq experiments to assay global populations of mRNAs. Datasets from 20 studies, most using high-throughput sequencing, were compiled and analyzed using a common pipeline allowing for their comparison, and the identification of a core neurite transcriptome (von Kügelgen and Chekulaeva, 2020). The datasets covered a range of sample types including neuroblastoma lines, primary neurons, ESC- and iPSC-derived neurons of various subtypes, and DRG explants, across mouse, rat, and human. In most cases, compartmentalized culture was performed using devices such as transwell inserts, where cells sit on a membrane containing tiny pores through which neurites extend and grow along the lower membrane surface (Taylor et al., 2022; Taylor and Houart, 2024). In this way, transwell inserts enable the separate isolation of neurite tissue, which is likely mostly axons, with dendrites contributing approximately only 10% of the neurite population (Rotem et al., 2017; Nijssen et al., 2018). Several of the included datasets were generated from neuropil dissection from tissue sections, however, where dendrites are well represented. The integrated analysis revealed a common set of transcripts as the most abundant, a core conserved neurite transcriptome, dominated by mRNAs encoding ribosomal and cytoskeletal proteins, with mitochondrial and synaptic proteins also well represented (Table 1; von Kügelgen and Chekulaeva, 2020). Another way to characterize the neurite transcriptome besides mRNA abundance is by focusing on transcripts enriched in neurites compared to the soma, indicative of active localisation, suggestive of neurite-specific functions. While mRNAs encoding common axonal and synaptic markers were often abundant in neurites, they were not typically enriched (von Kügelgen and Chekulaeva, 2020). 61 mRNAs were consistently neurite-enriched across datasets, mostly encoding ribosomal proteins. Many of these transcripts were shown by other studies to associate with ribosomes in neurites indicating their local translation (Table 1).

**TABLE 1:** Most abundant transcripts within the core neurite transcriptome identified from analysis of many neurite datasets, and whether they have been reported to undergo local translation.

Gene name	Function	Reported neurite ribosome association
*Actb*	Cytoskeleton	Yes
*Tpt1*	Outgrowth formation, mitochondrial regulation	Yes
*Rpl4*	Ribosomal protein	Yes
*Ybx1*	RNA binding protein	No
*Rps12*	Ribosomal protein	Yes
*Rps8*	Ribosomal protein	Yes
*Atp5b*	Mitochondrial function	Yes
*Ywhae*	Outgrowth formation	Yes
*Rpl6*	Ribosomal protein	No
*Npm1*	Nuclear protein; ribosome associated	Yes
*Map1b*	Cytoskeleton	No
*Fau*	Ribosomal protein	Yes
*Calm1*	Calcium regulation	Yes
*Rps3a1*	Ribosomal protein	Yes
*Kif5c*	Synaptic function	Yes
*Gap43*	Outgrowth formation, synaptic function	No
*Kif5a*	Axonal transport	Yes
*Park7*	Oxidative stress protection	Yes
*Arl3*	Membrane trafficking	No
*Vdac3*	Mitochondrial regulation	No
*Eef1a1*	Translation machinery	Yes
*Actg1*	Cytoskeleton	Yes
*Eef2*	Translation machinery	Yes
*Rplp1*	Ribosomal protein	Yes
*Rpl23*	Ribosomal protein	Yes

Adapted from von Kügelgen and Chekulaeva (2020).

Transcriptomic variation in neurites owing to different sample types was unclear, possibly due to neurite populations comprising mixtures of axons and dendrites, and added heterogeneity, such as multiple neuron sub-types being represented (von Kügelgen and Chekulaeva, 2020). Also, primary neuron cultures likely contain some glia. Clear signatures were also unidentifiable for pre- or post-synaptic markers, possibly due to the maturity stage of neurites. Alternatively, the ratio of axons to dendrites present may not favor the formation of mature synapses in large quantities. Such findings highlight the importance of obtaining pure neuron and neurite type populations to explore questions of local transcriptomic diversity.

Other studies that have focused on characterizing specifically either axonal or dendritic transcriptomes have provided subcellular compartment-specific and temporal-related insights. Axons from embryonic and adult rat DRG sensory neurons assayed by microarray, identified significant differences in the pools of mRNAs between these stages (Gumy et al., 2011). Similar numbers of mRNAs were present with substantial overlap in mRNA identity. At both stages, axons were enriched for mRNAs encoding ribosomal and mitochondrial proteins. Those uniquely enriched in embryonic axons encoded proteins involved in axon guidance and growth, whilst those uniquely enriched in adult axons encoded those involved in inflammation and immunity. In a later study, RNA-Seq from embryonic mouse DRG sensory axons revealed a high degree of similarity across species, identifying 80% of genes detected in the embryonic rat DRG axons (Minis et al., 2014), as well as detecting many more. Gene ontology (GO) categories for mRNAs enriched in this dataset included translation, in line with the rat study, and other categories including sequence-specific DNA binding, extracellular matrix, and immune response (Minis et al., 2014). While DNA binding terms may be initially surprising, this reflects the known axonal localisation of transcripts encoding classically nuclear proteins, including transcription factors thought to mediate axon-to-nucleus signaling (Ji and Jaffrey, 2014; Twiss and Merianda, 2015). Such axonal localisation of nuclear proteins and their mRNAs has been reported by many studies since, including *in vivo* (Alon et al., 2021).

In line with the observations from adult rat DRG axons (Gumy et al., 2011), RNA-Seq on axoplasm from adult rat ventral root motor axons revealed enrichment in GO terms associated with translation, mitochondria, and the cytoskeleton (Farias et al., 2020). Mitochondrial and ribosomal genes also dominate enrichments in human iPSC (hiPSC)-derived motor neurons grown in transwell inserts, where axons strongly dominate the neurite population (Maciel et al., 2018), and in mouse embryonic motor axons following culture in microfluidic chambers (Briese et al., 2016), respectively.

Laser capture and microdissection of specifically the growth cones of retinal ganglion cell (RGC) axons in mouse and *Xenopus laevis* revealed a surprisingly large number of mRNAs belonging to similar functional categories including protein synthesis, oxidative phosphorylation, and signaling. Moreover, mRNA repertoire in growth cones was shown to be regulated dynamically with age and become increasingly complex with time as it advances along the retinotectal pathway (Zivraj et al., 2010). Another study identified enrichment primarily of transcripts containing the non-canonical 5′ TOP (5′ termini oligopyrimidine) motif in RNA-Seq from just the growth cones of axons *in vivo* (Poulopoulos et al., 2019). This motif is found specifically in transcripts encoding ribosomal proteins and translation initiation factors, and acts as an ON/OFF switch controlling translation through its direct responsiveness to mTOR. By this mechanism, the authors speculate that 5′ TOP transcripts enriched in the growth cone may be translated upon mTOR signaling in response to target-derived growth signals, driving axonal growth.

Studies focused on elucidating dendrite-specific transcriptomes have often taken single cell approaches owing to difficulties in isolating dendrite tissue from somas (Middleton et al., 2019; Perez et al., 2021). Single cell RNA-Seq (scRNA-Seq) analysis of mouse primary hippocampal neurons identified dendrite enrichment of GO terms related to the ribosome and mitochondria, including ribosomal subunits, mitochondrial membrane, and respiratory chain complex (Steward, 1997; Middleton et al., 2019).

Early *in situ* hybridisation studies indicated that dendrites of different neuronal sub-types contain distinct mRNAs (Steward, 1997; Eberwine et al., 2001). Comparison of glutamatergic and GABAergic rat hippocampal interneurons following scRNA-Seq identified easily discernible cell type-specific transcriptomic differences between somas (Perez et al., 2021). *Map1a* and *Calm1* were the most abundant mRNAs in dendrites of both glutamatergic and GABAergic neurons. While transcriptomic variation across dendrites was more subtle, some sub-type specificity was observed in those from different GABAergic neuron types,

At the sub-dendritic level, mouse hippocampal pyramidal neurons observed *in situ* following expansion microscopy, showed differential distribution of mRNAs in spines compared with adjacent dendrite (Alon et al., 2021). The most abundant transcripts in spines were *Shank1, Adenyl cyclase1* and *Kif5a*, specifically localized here along with *Map1a* and *Map2a*. *Camk2a* and *Ddn* were enriched in dendrites compared with spines and cell bodies. Such data indicates additional layers of compartmentalisation.

Numerous studies have focused on the isolation and dissection of the transcriptomes of pre- and post-synapses. Indeed, RNA-Seq on synaptoneurosomes purified from the forebrains of 10-week-old mice revealed dominance of mRNAs pertaining to cellular compartment ontology terms including membrane, synapse, neuronal projection, and post-synaptic density, with biological process ontology terms including transport, cell adhesion and long-term synaptic potentiation (Simbriger et al., 2020). Similarly, synaptosomes from 3-month-old mouse hippocampus revealed enrichment for synapse-related ontologies, with KEGG-pathway analysis identifying the strongest enrichments in glutamatergic synapses, cAMP signaling and long-term potentiation, as well as presence of terms linked to mitochondrial function (Epple et al., 2021). Mature mouse forebrain synaptosomes enriched for vGLUT1+ pre-synaptic terminals, reflecting excitatory synapses, versus a non-purified population of synaptosomes and neurite material, identified 468 enriched transcripts dominated by GO terms including pre-synaptic active zone and ribosomal proteins (Hafner et al., 2019). The most enriched transcripts within the group included known pre-synaptic (*Stx6, Bsn, Rims1-3*) and signaling molecules (*Sergef, Rapgef4*). Transcripts less well represented in the vGLUT1+ synaptosomes compared with the general population included many coding for GABA and AMPA families - post-synaptic and dendritic components.

In summary, the studies described characterizing the transcriptomes of either a neurite mix, or exclusively axons or dendrites, identify overwhelming enrichment of mRNAs encoding factors associated with translation. These include constituent ribosomal proteins, and translation initiation and elongation factors. Such findings are intriguing given that ribosome production classically occurs in the nucleolus. Indeed, recent studies, including some in neurites, have reported that ribosomes are locally remodeled through incorporation of newly synthesized proteins, facilitating specialization or repair (Mathis et al., 2017; Shigeoka et al., 2019; Fusco et al., 2021). Future studies aimed towards dissecting ribosomal specificity underlying mRNA translation, and local changes to ribosomal makeup, will likely shed new light on the mechanisms by which local transcriptomes replenish and shape the neurite proteome. Mitochondria-related ontologies are also well represented across neurite types, reflecting their high metabolic demand. It is perhaps surprising that membrane and signaling proteins are not more dominant, however mRNA copy number often does not directly correlate with the number of proteins produced (Edfors et al., 2016; Zappulo et al., 2017). The mRNAs found enriched in pre- and post-synapses are highly specialized based on the functions of these compartments and the proteins found within them. It will be intriguing to see if there are additional sub-compartments within axons and dendrites that serve as hubs for specific mRNA pools. Indeed, interaction with different subcellular organelles within neurites can be indicative of their fate or translational status (see below).

We will now discuss the fates of localized mRNAs in both axonal and dendritic arbors as well as synaptic compartments.

### Fates and functions

#### Local translation

**FIGURE 2:**
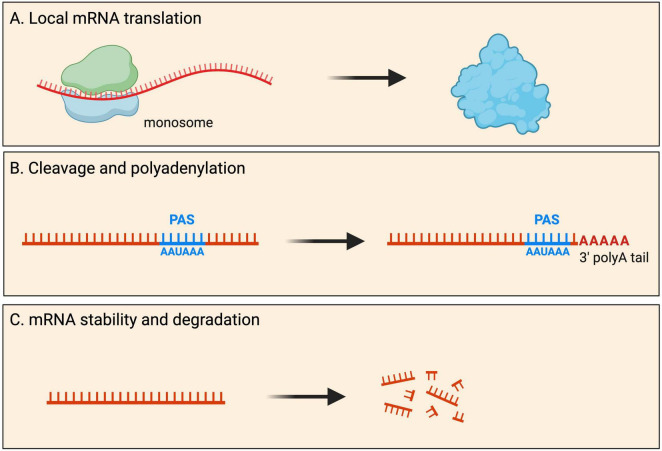
Fates of localized mRNAs in neurons. mRNA transcripts are transported into neurites within RNA granules. Within such local compartments mRNAs have been shown to undergo processing that includes: **(A)** mRNA translation for the local production of new proteins upon demand; **(B)** cleavage and polyadenylation of the 3′ UTR at the polyadenylation signal (PAS); and **(C)** mRNA stability as well as degradation.

Most mRNAs are transported to neurites within RNA granules, which are dynamic, membrane-less cellular structures that contain mRNA molecules and various proteins (Dalla Costa et al., 2021). Interestingly, recent imaging experiments showed that the dynamics of endogenous RNA granules correlate with new branch emergence and branch stabilization (Wong et al., 2017), indicating that localized mRNAs play a role in the formation and stabilization of neural connections. Traditionally, protein synthesis was thought to occur exclusively in the soma cytoplasm. However, it has become increasingly evident that local mRNA translation can occur, and is widespread, at specific subcellular locations within neurons. During local protein synthesis, mRNA molecules are translated into proteins near the site they are required. Such local protein synthesis sites range from axonal and dendritic branch points to developing and mature pre- and post-synaptic compartments, as well as near cellular organelles (Figure 2). It is thought that up to half of the proteome in neurites has local protein synthesis being the predominant source (Zappulo et al., 2017; Glock et al., 2021).

Within axons, dendrites and synaptic compartments, an increasing number of studies have begun to reveal the importance of associations between organelles and ribonucleoprotein (RNP) complexes for local translation (Pushpalatha and Besse, 2019; Vargas et al., 2022). Indeed, RNA-bearing Rab7a late endosomes were found to pause on mitochondria along RGC axons, facilitating translation of mRNAs encoding mitochondrial proteins (Cioni et al., 2019). This mode of local translation was shown to be important for mitochondrial function and axonal viability. In another study, tethering of certain transcripts to axonal mitochondria has been shown to be important for their translation to maintain the mitophagy pathway (Harbauer et al., 2022). In a late-endosomme independent manner, *PINK1* mRNAs require tethering to the mitochondrial outer membrane by Synaptojanin2 (SYNJ2), for their transport and translation (Harbauer et al., 2022). Intriguingly, translation of the PINK1 mitochondrial targeting sequence was also required for such transport, suggesting a local translated peptide was essential for the localisation of its own transcript to neurites. Other studies have identified important roles for the endoplasmic reticulum (ER) in local translation. It was recently shown that ribosomes associate with ER upon activation of local translation in motor axonal growth cones following their stimulation with brain-derived neurotrophic factor (BDNF) (Deng et al., 2021). It is likely that these ribosomes translate membrane and secreted proteins, classically translated at the rough ER, which was not known to occupy axons prior. Another study also showed ribosomes contacting ER tubules in a translation-dependent manner, in a process facilitated by the axonal ribosome/mRNA receptor P180/RRBP1 (Koppers et al., 2022). Future studies will reveal the contribution of other organelles to local translation in neurites, and identify which mRNAs require specific organelles for the process.

Global pools of locally translated mRNAs in neurites have been revealed by studies using novel ribosome capturing and RNA sequencing techniques. One such technique was the development of axon-TRAP-RiboTag (Shigeoka et al., 2016), utilizing a mouse line harboring a modified allele of the ribosomal Rpl22 protein fused to a HA tag (Rpl22-HA), induced by the action of a Cre recombinase (Sanz et al., 2009). Using a RGC-specific Cre line, full-length mRNAs pulled-down with HA-tagged ribosomes revealed the local translatome within RGC axons at multiple stages (Shigeoka et al., 2016). This identified a dominance of mRNAs encoding proteins involved in vesicle-mediated transport and calcium-mediated signaling. Ribosome immunoprecipitation approaches have also been used to identify locally translated transcripts in dendrites isolated from adult mouse hippocampus, revealing a dominance of mRNAs encoding translation and cytoskeletal proteins (Ainsley et al., 2014). Transcripts encoding nuclear proteins, including histones, were also observed, as in axons (see above section on mRNA diversity).

An alternative method for determining which proteins are actively translated locally is ribosome footprinting/profiling (Ingolia et al., 2012). Also known as Ribo-Seq (ribosome sequencing) or ART-Seq (active mRNA translation sequencing), it provides a snapshot, revealing RNA fragments/“footprints” protected by ribosomes caught during active translation. To identify and quantify the transcriptome and translatome in cell bodies (somata) as well as dendrites and axons, a recent study performed simultaneous RNA-Seq and Ribo-Seq from micro-dissected hippocampal rodent brain slices (Glock et al., 2021). The study led to the identification of more than 800 mRNAs whose dominant source of translation is the neuropil, suggesting that many axonal/dendritic and synaptic proteins arise mostly from local translation (Glock et al., 2021). But how do these localized mRNAs undergo local protein synthesis? During translation in the soma, multiple ribosomes can occupy an individual mRNA (a complex called a polysome), resulting in the simultaneous generation of multiple copies of the encoded protein. A recent study showed that monosomes (single ribosomes), as opposed to polysomes, are the predominant ribosome population in neuronal processes (Biever et al., 2020). Indeed, measuring ribosome density on transcripts in synaptic neuropil, revealed monosomes predominantly elongate key synaptic transcripts in both dendritic and axon terminals (Biever et al., 2020). One possible explanation for the difference between somatic and local mRNA translation that could explain the high abundance of monosomes in the neuropil, is the production of a more diverse set of proteins from a limited pool of available ribosomes found at synapses (Ostroff et al., 2017).

Local protein synthesis is a highly regulated process, with most local transcripts not translated by default. Some of the most abundant transcripts in neurites seemingly do not associate with ribosomes (Table 1; von Kügelgen and Chekulaeva, 2020). Studies comparing the mRNA species constituting local transcriptomes and those associating with ribosomes, reveal that only specialized subsets of transcripts become translated, in a spatio-temporal fashion. Furthermore, ribosomal footprinting data from synaptoneurosomes reported that mRNAs undergoing translation were associated with different ontologies (mitochondrial and extracellular matrix and exosome proteins) to those generally dominant (see above in mRNA diversity section) (Simbriger et al., 2020).

How dynamic is the local translatome? Certain mRNAs encoding regulators of protein and energy homeostasis, and those associated with vesicle-mediated transport and calcium-mediated signaling are translated regardless of developmental stage (Shigeoka et al., 2016). Other mRNAs are dynamically regulated during development and maturation, suggesting that local translation plays an important role in the homeostasis of neurites. The translatome of younger axons was enriched for GO terms such as neuron projection morphogenesis (Shigeoka et al., 2016). Contrastingly, the adult axonal translatome was found to have strong links to axon survival, neurodegenerative disease, and neurotransmission, with key components of the trans-SNARE complex, which mediates neurotransmitter exocytosis, being highly translated in mature axons (Shigeoka et al., 2016). The findings indicating that axonal mRNA translation persists in adult CNS axons were intriguing because it has been controversial whether mature CNS axon terminals can synthesize proteins at all, partly because of early studies detecting few or no ribosomes in mature axons (Koenig et al., 2000). Therefore, these findings showed conclusively a unique adult local translatome is present in mature axons, whose main role is likely to be the regulation of synapse function. In contrast, local translation of transcripts involved in axonal and dendritic elongation, branching, pruning, synaptogenesis, and synaptic transmission occurs developmentally (Shigeoka et al., 2016; Biever et al., 2020), indicating the process has an equally crucial role in regulating neuronal connectivity and synaptic plasticity. Indeed, local translation is thought to enable neuronal cells to respond to signals from the environment. For instance, extracellular cues (e.g., Netrin-1, BDNF, Sema3A) were found to differentially influence axonal synthesis of multiple proteins in a cue-specific and temporally dynamic manner. Interestingly, the synthesis of proteasomal subunits (α and β type), some ribosomal proteins, histones, and methyltransferases is differentially regulated in response to such cues (Cagnetta et al., 2018). The significance of local mRNA translation in neurite growth is supported by functional experiments in *Xenopus laevis* RGC axons (Wong et al., 2017). Here, local protein synthesis was found to be essential for proper axon arbor formation *in vivo*, as inhibition of local translation or knockdown of local β-actin synthesis caused a marked reduction in axon branching dynamics and arbor complexity (Wong et al., 2017).

Local protein synthesis is also required for synaptic plasticity. At synapses, local protein synthesis was found to be differentially recruited to drive compartment-specific phenotypes that may underlie different forms of plasticity (Hafner et al., 2019). Evidence for a role of local translation in synaptic plasticity comes from a study utilizing dissociated rat hippocampal neuron cultures. During basal synaptic transmission, the amount of locally synthesized proteins detected at a synapse was correlated with its level of ongoing spontaneous activity. Plasticity induced by single-spine stimulations or by a global activity manipulation resulted in a significant increase in local protein synthesis (Sun et al., 2021). Similarly, depolarization of primary cortical neurons caused rapid reprogramming of dendritic protein expression (Hacisuleyman et al., 2024).

Many locally translated transcripts interact with RNA binding protein (RBPs) through sequences found within the non-coding untranslated regions (UTRs) (Andreassi et al., 2018). Such interactions have been shown to regulate local translation. A well-known negative regulator is Fragile X Messenger Ribonucleoprotein (FMRP), which has been shown to interact with the coding region and 3′ UTR of many mRNA transcripts encoding pre- and post-synaptic proteins, many of which were found to be linked to autism (Darnell et al., 2011; Ouwenga et al., 2017). These RNA-protein interactions repress the local translation of transcripts, with dendritic (Hale et al., 2021) and axonal (Jung et al., 2023) FMRP target mRNAs showing increased ribosome association in *Fmr1* knockout mice. RBFOX1, which regulates the splicing of many exons in neurons, binds to the 3′ UTR of cytoplasmic mRNA targets involved in cortical development and autism to increase their stability and local translation (Lee J. A. et al., 2016). Another positive regulator of local protein synthesis is PTBP2, which was shown to interact with the 3′ UTR of *Hnrnpr* mRNA, mediating the association of *Hnrnpr* with ribosomes in a translation factor eIF5A2-dependent manner (Salehi et al., 2023). Indeed, local synthesis of hnRNPR protein is strongly reduced when PTBP2 is depleted, leading to defective motor axon growth (Salehi et al., 2023).

It has been hypothesized that longer UTR sequences may permit a greater number of binding motifs for post-transcriptional regulation, including increased local protein synthesis (Andreassi and Riccio, 2009). Thus, an additional mechanism for regulating local protein synthesis could be through alternative splicing, such as the production of transcripts with alternative last exons (ALEs), and thus distinct 3′ UTRs. Indeed, transcripts with ALEs are disproportionately found in neurites (Taliaferro et al., 2016) undergoing local mRNA translation (Ouwenga et al., 2017). Moreover, cis-regulatory elements generated by alternative splicing at 5′ and 3′ UTRs have been shown to promote axonal mRNA translation (Shigeoka et al., 2016). Therefore, alternative splicing at the UTRs could influence the ability of transcripts to be locally translated. Control of mRNA translation in neuronal subcellular compartments is discussed in more detail elsewhere (Cagnetta et al., 2023).

#### Cleavage and polyadenylation

3′ UTRs are involved in many aspects of mRNA metabolism, including intracellular localisation and translation. Surprisingly, global mapping of 3′ end regions indicated that ∼75% of mammalian genes contain more than one polyadenylation (poly(A)) site (PAS), giving rise to multiple 3′ UTRs (Proudfoot, 2011; Tian and Manley, 2013; Gruber and Zavolan, 2019). There is remarkable variation in PAS and 3′ UTR length between tissues, with neurons characterized by significantly longer 3′ UTRs (Miura et al., 2013). During neuronal development, many genes are subjected to 3′ UTR and/or poly(A) lengthening (Miura et al., 2013; Kiltschewskij et al., 2023), suggesting this constitutes an important mechanism of post-transcriptional mRNA regulation associated with neuronal differentiation. The process is thought to be a mechanism that serves unique post-transcriptional regulatory needs of transcripts in neurons e.g., transcript localisation, stabilization, and local protein synthesis regulation (Miura et al., 2014).

Although 3′ end cleavage and polyadenylation predominantly occur in the soma, evidence for local processing of alternative 3′ UTR isoforms has also been observed in axons and dendrites (Figure 2). Within neurites, many local mRNA transcripts have long 3′ UTRs and have significantly longer half-lives than somata-enriched isoforms (see mRNA stability and degradation section below) (Tushev et al., 2018). Interestingly, these 3′ UTR isoforms can be significantly altered by neuronal activity, with elevated activity resulting in significant shortening of neuropil-localized 3′ UTR isoforms (Tushev et al., 2018). Although most 3′ UTR plasticity was found to be transcription-dependent, evidence for transcription-independent changes was also reported (Tushev et al., 2018), hypothesized to arise from altered stability, trafficking of 3′ UTR isoforms between soma and neuropil, or local remodeling of 3′ UTRs by shortening or lengthening. Direct evidence for local cleavage and polyadenylation comes from work on rat sympathetic neurons showing that axons and cell bodies express distinct pools of 3′ UTR isoforms (Andreassi et al., 2021). Axon-specific short 3′ UTR isoforms of *IMPA1*, *Maoa*, and *Sms* are generated through a process of 3′ UTR cleavage and polyadenylation in axons. This local processing generates translatable isoforms necessary for maintaining the integrity of sympathetic neuron axons (Andreassi et al., 2021). Local cleavage and polyadenylation are further supported by a recent study showing that exposure of sympathetic neurons to Nerve Growth Factor (NGF) or Neurotrophin 3 (NT-3) induces the localization of distinct 3′ UTR isoforms to axons, including short 3′ UTR isoforms found exclusively in axons (Luisier et al., 2023). These observations support a model whereby long 3′ UTR isoforms associate with RBP complexes in the nucleus and, upon reaching the axons, are remodeled locally into shorter isoforms.

A key factor controlling non-nuclear polyadenylation is cytoplasmic polyadenylation binding protein (CPEB), an RBP with strong association for the cis-acting cytoplasmic polyadenylation element (CPE) residing in 3′ UTRs of target mRNAs. CPEB regulates poly(A) tail length by interacting with deadenylating enzymes as well as noncanonical poly(A) polymerases. Many of the components of the cytoplasmic polyadenylation machinery have been found at post-synaptic sites of hippocampal neurons, including CPEB, the scaffold protein Symplekin, the deadenylase poly(A) ribonuclease (PARN), the noncanonical poly(A) polymerase germ line defective 2 (Gld2), and CPEB-interacting factor neuroguidin (Ngd) (Jung et al., 2006; Udagawa et al., 2012; Swanger et al., 2013). The decision whether CPEB binds a deadenylating enzyme (e.g., PARN) favoring short poly(A) tails and translational dormancy, or noncanonical poly(A) polymerases (e.g., Gld2) favoring elongated poly(A) tails and translation, depends on its phosphorylation (Barnard et al., 2004, 2005). Generally, synaptic stimulation promotes phosphorylation, which in turn stimulates poly(A) tail lengthening and local translation (Ivshina et al., 2014).

The cytoplasmic polyadenylation machinery locally acts to bidirectionally regulate mRNA-specific translation and plasticity at hippocampal synapses in response to synaptic transmission, with the poly(A) tail of 102 mRNAs shortened following depletion of Gld2 (Udagawa et al., 2012). One such local transcript is *NR2A* (or GluN2A) mRNA, encoding an NMDA receptor subunit, which contains CPEs in its 3′ UTR, has a short poly(A) tail and is translated inefficiently (Udagawa et al., 2012). *NR2A* RNA is bound by CPEB, which in turn is associated with PARN, Gld2, Symplekin, and Ngd. However, because Ngd is also bound to the cap binding factor, eIF4E, translation is blocked at initiation. NMDA receptor activation was found to promote phosphorylation of CPEB, expulsion of PARN from the RNP complex, and Gld2-catalyzed poly(A) lengthening of *NR2A* mRNA (Udagawa et al., 2012). This local polyadenylation is thought to displace Ngd from eIF4E, the binding of eIF4G to eIF4E, resulting in enhanced translation of NR2A mRNA and membrane insertion of NMDA receptors in dendrites (Swanger et al., 2013). These findings indicate that local polyadenylation has an important role in the activity-dependent synthesis, and NMDA receptor surface expression during synaptic plasticity. Indeed, depletion of CPEB or one of the noncanonical poly(A) polymerases from the mouse hippocampus results in a deficit in long term potentiation (LTP) and increase in long-term depression (LTD) (Zearfoss et al., 2008; Udagawa et al., 2012; Mansur et al., 2021).

#### Stability and degradation

Neurite-localized transcripts have longer half-lives than somata-enriched isoforms, with average half-lives of mRNAs recorded as 4.8 h and 3.7 h, in neurites and soma cytoplasm of primary cortical neurons, respectively (Tushev et al., 2018; Loedige et al., 2023). The stability and degradation of mRNAs in neurites are crucial for various neuronal functions, including neurite outgrowth and synaptic plasticity. Neurites are an integral part of neuronal communication, and the regulation of mRNA stability in these structures plays a key role in shaping neuronal responses (Figure 2). Several factors contribute to the regulation of mRNA stability and degradation in neurites. Below, we will review the evidence that supports a complex network of RNA-protein interactions underpinning the dynamics of mRNA stability and degradation in neurites.

How do longer 3′ UTRs link with increased stability of local mRNAs? It was postulated that alternative 3′ UTRs have novel and repeated regulatory motifs that might help establish localisation to distal regions of the dendrite or axon (Tushev et al., 2018). RBPs are increasingly found to be essential for transcript stability. Such RBPs, including FMRP, STAUFEN2 (STAU2), and TAR DNA-binding protein 43 (TDP-43), are often found to be associated with their mRNA targets in distal dendritic and axonal branches and synapses (Ortiz et al., 2017; Sharangdhar et al., 2017; Chu et al., 2019). Examples also include many RNA splicing regulators that localize in a bimodal fashion to both the nucleus and neurites, where they facilitate RNA metabolism. Such regulators include the Muscleblind proteins, which regulate alternative splicing in the nucleus (Pascual et al., 2006; Konieczny et al., 2014) and the correct localisation of mRNAs in neurons (Wang et al., 2012; Hildebrandt et al., 2023). Evidence from the nematode *Caenorhabditis elegans* (*C. elegans*) indicates that Muscleblind-1 (MBL-1) binds to mRNA transcripts encoding microtubule proteins to regulate their stability. Indeed, microtubule stability in sensory neuron axons is compromised in *mbl-1* mutants due to reduced levels of α-tubulin and β-tubulin (Puri et al., 2023). Another well-known splicing regulator also involved in RNA stability is SNRNP70, a core spliceosome protein. SNRNP70 was found to localize to cytoplasmic RNA granules and associate with mRNA transcripts, controlling their axonal trafficking and stability in zebrafish motor neurons, ultimately regulating neuromuscular connectivity (Nikolaou et al., 2022).

The longer half-lives of localized transcripts can also be explained by a lack of destabilization elements. Evidence suggests that neurite-localized mRNAs are depleted of destabilizing elements (Loedige et al., 2023). Such sequences include AU-rich elements (AREs), and those that promote m^6^A (N^6^-methyladenosine) modifications which induce mRNA degradation. It was shown that high mRNA stability is both necessary and sufficient for localisation to neurites, with depletion of mRNA-stabilizing proteins ELAVL and LARP1 interfering with transcript localisation to neurites. Also, alleviation of m^6^A-dependent mRNA degradation by depletion of YTHDF, or removal of destabilizing AREs, were sufficient to increase the stability of transcripts and shift these toward neurites (Loedige et al., 2023).

The most extensively studied mechanism for RNA degradation is by nonsense-mediated RNA decay (NMD), a cellular surveillance mechanism that recognizes and degrades mRNAs containing premature termination codons (PTCs) or nonsense mutations. NMD is a crucial quality control mechanism in eukaryotic cells, ensuring the removal of faulty transcripts and maintaining the integrity of the cellular proteome. The NMD pathway involves a series of proteins and complexes that recognize PTCs and facilitate mRNA degradation. Key components include UPF1, UPF2, and UPF3, which form the core NMD complex. These proteins interact with the exon junction complex (EJC) and other factors to initiate mRNA degradation (Lykke-Andersen and Jensen, 2015). Although NMD is initiated as soon as a PTC is detected in the nucleus, evidence suggests that the pathway can also operate locally to regulate neurite outgrowth, axon guidance and synaptic plasticity through the degradation of selected mRNA isoforms containing NMD-inducing PTCs (see IR RNAs section).

In the hippocampus, the NMD pathway operates within dendrites to regulate synaptic function and plasticity by increasing Glutamate receptor, GLUR1, surface levels (Notaras et al., 2020). UPF2 was shown to promote local synthesis of GLUR1 in dendrites through local NMD-mediated degradation of *Arc* and *Prkag3* mRNAs, whose proteins negatively influence local translation (Notaras et al., 2020). This observation demonstrates that local translation is regulated by mechanisms that control mRNA degradation in dendrites. In addition to its canonical targets, NMD may also degrade mRNAs that do not carry identifiable NMD-inducing features (He and Jacobson, 2015), however, the mechanisms by which NMD recognizes its atypical targets remain unclear. It is also possible that NMD components could act independently of mRNA degradation to promote local protein synthesis. Indeed, UPF1 was found to regulate synaptic plasticity in hippocampal neurons by facilitating the transport and translation of mRNAs through its association with STAU2 (Graber et al., 2017).

## Intron-retaining RNAs (IR RNAs)

### Diversity in axons, dendrites, and synapses

Introns are sections of DNA within genes that intersperse exons. Generally considered non-coding sequences, they are typically spliced from pre-mRNAs co-transcriptionally. Sometimes, however, one or multiple introns may be retained in the mature transcript (Grabski et al., 2021). In recent years, the development of pipelines to identify intron retention events in high-throughput sequencing datasets, has revealed it to be a more common phenomenon than previously thought, and more widespread in neurons compared to other tissues (Braunschweig et al., 2014; Jacob and Smith, 2017; Middleton et al., 2017). Intron retention has mostly been considered in a nuclear context, either as a mechanism of inducing transcript degradation, thereby driving gene downregulation, or to detain transcripts in the nucleus, delaying their export until required. More recently, however, many intron-retaining (IR) mRNAs have been reported to localize and even become enriched in the cytoplasm and neurites (Figure 1), pointing towards functional roles for local IR isoforms. Below, we outline the key studies characterizing intron-retaining transcript populations in axons, dendrites, and synapses.

Early studies detected IR mRNAs in cultured embryonic rat hippocampal neuronal dendrites following reverse transcription of extracted mRNA and PCR amplification, and by microarray analysis and *in situ* hybridization (Bell et al., 2008; Buckley et al., 2011). Such IR transcripts pertained to genes encoding proteins such as synaptic proteins, ion channels, RBPs (inc. splicing factors), and translation factors (Buckley et al., 2014; Luisier et al., 2018). More recent studies have leveraged high-throughput sequencing approaches to more thoroughly identify and quantify IR transcripts in neurites. Primary rat hippocampal neurons cultured in transwell inserts, enabling the isolation of neurites, identified 428 neurite-enriched retained introns (Saini et al., 2019). In another study, mouse embryonic motor neurons cultured in microfluidic chambers revealed intronic sequences to be detected more strongly in axons compared to the somatodendritic compartment, likely representing IR transcripts (Briese et al., 2016). Many retained introns have also been reported in zebrafish neurites following primary culture of larvae-derived neurons in transwell inserts (Taylor et al., 2022). The same study also revealed dramatic neurite-specific increases in IR transcripts in absence of the neuronal-enriched splicing factor, SFPQ, identifying the protein as a key regulator of neurite intron retention.

Little is known regarding IR RNA localisation to synapses; partly due to a lack of RNA-Seq analyses mining for events from synapse-specific samples. However, *CamKIIa* intron-16-retaining RNAs were identified in synaptoneurosomes isolated from mouse primary cortical neurons and adult cortical tissue, and their levels were shown to decrease upon stimulation with BDNF or N-methyl-D-aspartate (NMDA) (Ortiz et al., 2017). These findings suggests a wider array of IR transcripts may be detected at synapses in future RNA-Seq analyses.

Thus far, most neurite-localized IR transcripts have been detected in cultured neurons. Recent data confirms localisation of such transcripts in tissue, in distal dendrites of hippocampal neurons imaged *in situ* following expansion microscopy combined with long-read sequencing (Alon et al., 2021). This includes *Grik2*, a glutamate ionotropic receptor kainate subunit implicated in excitatory glutamatergic neurotransmission.

In RNA-Seq datasets from neurite samples, often multiple introns within the same gene show reads mapping to them. However, it is unclear whether such introns are retained together in the same transcript isoform, or retained individually in distinct isoforms. This is due to the short-read lengths used in standard RNA-Seq experiments. The advent of third-generation long-read sequencing datasets will provide new insights that address this question. Multiple introns retained in a single isoform suggests even greater complexity in intron retention regulation, and the functions of IR mRNAs.

### Fates and functions

#### Local translation and degradation

**FIGURE 3:**
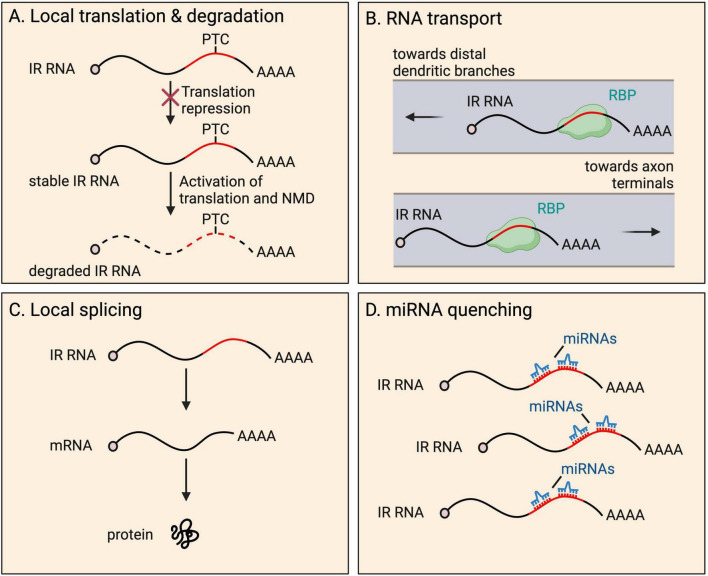
Fates and functions of intron-retaining (IR) RNAs in neurites. IR RNAs have several fates and functions within distal parts of neurons, including: **(A)** local translation and subsequent degradation due to presence of a premature termination codon (PTC), a process that provides tight temporal and spatial control of protein expression; **(B)** RNA granule organization and RNA transport toward distal dendritic and axonal regions; **(C)** local splicing to boost the pool of translatable fully spliced mRNAs; and **(D)** miRNA quenching through the harboring of miRNA recognition motifs.

IR transcripts are thought to rarely serve a coding function. Retained introns frequently insert PTCs into transcripts, expected to activate transcript degradation by NMD upon the pioneer round of any translation. A well-known example of local translation of an IR mRNA occurs in the developing spinal cord (Chen et al., 2008; Colak et al., 2013). Here, commissural axons are initially attracted to the ventral midline and, upon crossing, become repulsed. Such axon guidance depends on the interaction between axon membrane receptors (Robo proteins) and proteins of the extracellular matrix (Slit proteins) (Jaworski et al., 2010). Following transcription, *Robo3* transcripts are processed into either of two isoforms−fully spliced *Robo3.1* (no IR), and intron-26-retaining *Robo3.2* containing a PTC (Chen et al., 2008; Colak et al., 2013). Prior to reaching the ventral midline, *Robo3.1* mRNAs are translated, preventing activation of ROBO1 and ROBO2 that are present at low levels on axons, while *Robo3.2* transcripts are translationally repressed. Once the axon has been exposed to floorplate signals in the spinal cord midline, rapid translation of *Robo3.2* mRNA is triggered, producing a peptide with a distinct C-terminus compared to the peptide produced from *Robo3.1*. ROBO3.2 protein increases the ability of ROBO1 and ROBO2 to bind to Slit proteins, which in turn repels the axon from the midline area, allowing appropriate axon positioning (Chen et al., 2008). The ROBO3.2 C-terminus is composed of intron-encoded amino acid residues up to the PTC. As expected, *Robo3.2* translation also activates NMD of the transcript, however, this was shown to be functionally important, limiting production of the protein to the correct quantity (Figure 3). Blocking NMD in commissural neurons caused accumulation of *Robo3.2* mRNA and ROBO3.2 protein and disproportionate axon repulsion from the midline, indicating the physiological importance of NMD to ensure functionally relevant amounts of protein are synthesized. Thus, NMD drives tight temporal and spatial control of the expression of the protein (Colak et al., 2013).

Other examples of proteins from IR transcripts have also been described. SMN1 functions in spliceosome assembly, implicating it in the splicing process. A specific isoform, aSMN, produced from an mRNA retaining intron-3, is found in axons and is important for axonogenesis (Setola et al., 2007). The specific function/s of the shortened peptide are unclear. In another study, *Nxf1*, which encodes the nuclear export factor NXF1, produces a transcript that retains intron-10 and undergoes translation to produce the shortened protein isoform, sNXF1, detected in dendrites of rodent cortex (Li et al., 2016). Intron-10 contains a constitutive transport element, which requires NXF1 for nuclear export of the IR mRNA (Li et al., 2006). The authors report a high level of expression of sNXF1 in endogenous adult rodent brain suggesting either the IR transcript does not undergo NMD, or that it is expressed at very high levels.

The extent to which translation occurs more widely from neurite-localized IR mRNAs is unclear. Analyses of neurite ribosome profiling/footprinting data have not explored the extent to which reads map to introns, likely because proportionally they represent very few, owing to NMD activation. Given that PTCs are introduced at some point within introns, reads would be expected to map specifically to the 5′ of introns. However, retained introns could alternatively produce novel peptides by introducing novel translational start sites. An example of this has not yet been reported in neurites. Translation from sequences outside canonical coding regions such as introns, typically produces unstable proteins with hydrophobic tails, either targeted for degradation by the proteasome, or to the membrane (Kesner et al., 2023). However, more stable proteins may be produced from IR transcripts, where retention status is often conserved (Sorek and Ast, 2003; Galante et al., 2004; Buckley et al., 2014), and introns are more GC rich than non-retained introns (Braunschweig et al., 2014).

#### Transport and granule organization

Retained introns have also been shown to be important for RNA transport to neurites (Buckley et al., 2011; Ortiz et al., 2017; Figure 3). Many retained introns, including *Fmr1* intron-1, were shown to contain ID elements with motifs that were previously shown to regulate BC1 ncRNA localisation to dendrites (Buckley et al., 2011). *Fmr1* encodes FMRP, which localizes to the soma and dendrites, and is important for proper synaptic plasticity (Richter and Zhao, 2021). Reporters expressing *Fmr1* intron-1 ID elements exhibit dendrite localisation, and compete with endogenous IR transcript populations, resulting in altered distribution of the overall population of FMRP protein (Buckley et al., 2011). Mutations in the ID element dramatically reduced dendritic targeting of the reporters, indicating the importance of the sequence to achieve localisation.

STAU2 binds to retained intron-16 of *CaMKIIα*, required for dendrite localisation of transcripts in mouse hippocampal neurons (Ortiz et al., 2017). Intron-16 retention is conserved in human (Braunschweig et al., 2014) and rat (Buckley et al., 2011), suggesting it may have a conserved role. The authors investigated the fate of IR transcripts under different conditions. Blocking protein synthesis by cycloheximide treatment does not increase intron-16-retaining transcript expression when not undergoing synaptic stimulation, indicating the transcripts are not NMD targets under these conditions. Stimulation with BDNF or NMDA results in decreases in intron-16-retaining *CaMKIIα* transcripts, which was prevented by cycloheximide treatment, suggestive of translation-dependent degradation by NMD. However, given that overall transcript levels are unaffected by stimulation, one could also hypothesize that the intron-16-retaining portion are instead locally spliced (see Local splicing section below). CaMKIIα protein levels or isoform differences following stimulation were not investigated.

STAU2 has also been shown to be required for the transport of an IR *Calm3* mRNA, in dendrites of mature rat hippocampal neurons (Sharangdhar et al., 2017). In this case, the 5′ and 3′ exons flanking the intron are 3′ UTR, sequence classically associated with mRNA transport. Overall, STAU2 was found to strongly bind retained introns within the 3′ UTRs of 28 mRNAs, suggesting the protein similarly regulates the localisation of other transcripts.

Many questions remain regarding the nature of transport granules containing IR RNAs. One hypothesis is that IR transcripts act as the means of transport of fully spliced counterparts occupying the same granule. Such retained introns could also act as a scaffold/platform, binding relevant RBPs, facilitating time or activity sensitive RNA processing of neighboring spliced transcript counterparts. Alternatively, retained introns could act as a scaffold, binding RBPs to catalyze granule organization. Similar roles have been reported for 3′ UTR sequences (Ma and Mayr, 2018; Mayr, 2019).

#### Local splicing

The local splicing of IR mRNAs could provide a powerful means for the rapid expansion of the pool of translatable mRNAs when needed, or for local decisions to be made on whether to excise introns alone or with a neighboring exon to generate alternative protein isoforms on demand. Direct mechanistic evidence for endogenous local splicing has yet to be shown, and its possibility remains a controversial hypothesis in the field. Despite studies finding an increasing number of splicing factors localized to neurites, spliceosomes are huge and complex structures, and only a small portion of the snRNA and protein components have been detected at substantial levels (Poulopoulos et al., 2019). Below, we discuss the studies that have shown evidence supporting the possibility of local mRNA splicing (Figure 3).

One early study focused on the 6000-nucleotide long retained intron-16 in *Kcnma1* transcripts (Bell et al., 2008). Intron-16-retaining transcripts were estimated to form 10% of the total population of *Kcnma1* transcripts in rat hippocampal neuron dendrites. Targeting specifically the IR isoform with siRNAs was able to specifically reduce their pools. Significantly lower levels of KCNMA1, a calcium-activated BK channel protein, and perturbed neuronal firing properties were also observed. The authors hypothesized that intron-16 may be locally spliced in dendrites to increase the pool of translatable mRNAs. In a subsequent study by the same group, intron-17 of *Kcnma1* was also shown to be retained (Bell et al., 2010). STREX (stress axis regulated exon) is an alternative exon sitting immediately downstream of intron-17. The intron contains regulatory elements controlling the splicing of STREX in response to activity. Inclusion of the exon alters the activity of the channel the protein sits in. In the study, intron-17-retaining mRNAs were detected in dendrites, with the intron either retained alone or in combination with STREX. Knockdown of intron-17-retaining isoforms downregulates STREX-containing isoforms of KCNMA1, most prominently in dendrites, and also disrupts the burst firing abilities of hippocampal neurons. The authors suggested intron-17-retaining isoforms become spliced within dendrites, facilitating the production of STREX-containing KCNM1A. However, the mechanism by which any splicing event would occur is unclear and was not addressed in either study.

Intriguingly, an earlier study by the group indicated canonical splicing capabilities in dendrites of primary cultured rat hippocampal neurons, a process widely accepted as exclusively nuclear (Glanzer et al., 2005). U1 snRNA and splicing factors required for spliceosome assembly were detected by *in situ* hybridisation and immunohistochemistry, respectively. Dendrites were isolated from somas and transfected with the pre-mRNA splicing construct, chicken *δ-crystallin (cdc)* mRNA, consisting of a 257-nucleotide intron flanked by exons-14 and -15, with a FLAG sequence in-frame with exon-15. Spliced transfected mRNA was detected in 50% of experiments, with multiple splice junction variants clustering around the canonical donor and acceptor splice sites suggesting classic pre-mRNA splicing. FLAG epitope was also detected in dendrites, which was not possible without local splicing. Another more recent study suggesting canonical splicing occurring outside of the nucleus in neurons, identified that cytoplasmic pools of spliceosomal protein, SNRNP70, a core U1 snRNP component, rescue defects in alternative splicing events in *snrnp70* null zebrafish embryos (Nikolaou et al., 2022). Rescued events were enriched in genes associated with neuronal ontologies such as synaptic vesicle recycling proteins.

Although studies thus far have explored the possibility of canonical local splicing, the mechanism may be non-canonical, such as that described at the endoplasmic reticulum (ER) membrane during the unfolded protein response (UPR) (Back et al., 2006; Uemura et al., 2009). The accumulation of incorrectly folded proteins in cells causes ER stress and subsequent activation of the UPR to resolve the situation. This involves upregulated transcription of *XBP1*, mRNAs of which localize to the ER surface where a 26-nucleotide intron is excised by IRE1, inositol requiring kinase-1, which has endoribonuclease activity. The exposed mRNA 5′ and 3′ fragments are then ligated. Following non-canonical splicing the transcript undergoes translation producing a transcriptional activator of genes involved in the UPR. ER extends into axons and dendrites and could therefore similarly act as a platform for neurite splicing events (Öztürk et al., 2020).

#### miRNA quenching

A role in microRNA (miRNA) regulation has been suggested for retained introns in the cytoplasm of motor neurons (Figure 3). A recent study identified that a specific set of introns become transiently retained in the cytoplasm of neural precursor cells during lineage restriction of human iPSC-derived motor neurons (Petrić Howe et al., 2022). Intriguingly, these introns were enriched for 14 miRNA motifs. The authors showed that the IR transcripts are not targets for downregulation by miRNA binding. Conversely, reduced IR transcript expression led to increased expression of predicted miRNA target genes (a readout of miRNA activity). Such findings were not explained by changes in miRNA levels. The authors suggest the retained introns act as sponges, quenching miRNA binding and action on target mRNAs. Intriguingly, the reported retained introns were also enriched for binding capacity of miRNA regulatory proteins, including DROSHA and PUM2. However, loss of DROSHA did not affect levels of IR transcripts suggesting the protein does not process miRNAs from the introns. Regardless, it remains intriguing to hypothesize in other cases that miRNAs could be synthesized locally from introns. Thus far, processing of miRNAs from introns has only been observed in the nucleus (Westholm and Lai, 2011). While the study did not focus on neurite-localized IR transcripts, many miRNAs are known to localize to axons and dendrites (see ncRNAs section below), suggesting similar regulation could be present in neurites.

## Non-coding RNAs (ncRNAs)

**FIGURE 4:**
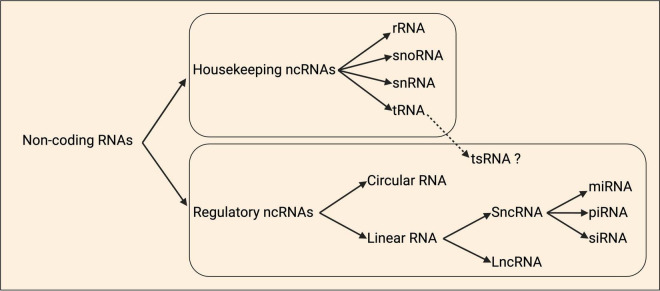
Schematic classification of non-coding RNAs. Non-coding RNAs (ncRNAs) are organized as housekeeping and regulatory ncRNAs. Housekeeping ncRNAs are divided into ribosomal (rRNA), small nucleolar (snoRNAs), small nuclear (snRNA), and transfer (tRNA). tRNA-derived small RNAs (tsRNAs) are a group of ncRNAs that are hypothesized to have regulatory roles. Regulatory ncRNAs include the circular and linear RNAs, and within the latter class there are the short ncRNAs (sncRNAs) and long ncRNAs (lncRNAs). The sncRNAs group is divided into the microRNAs (miRNAs), small interfering RNAs (siRNAs), and piwi-associated RNAs (piRNAs). Adapted from Baptista et al. (2021).

ncRNAs are diverse, and often loosely categorized either by size as short or long, or functionally based on whether they are housekeeping (tRNA, rRNA, snRNA, snoRNA) or regulatory (lncRNA, sncRNA including miRNA, circRNA) (Figure 4; Li et al., 2021; Mattick et al., 2023). Comparatively little is known regarding the true diversity amongst local ncRNAs at subcellular resolution in axons, dendrites, and synapses, including their relative abundance. However, data from motor axons identified that some of the most abundant localized transcripts are ncRNAs (Figure 1), including the rRNA, *Gm26924*, and *7SK* and *7SL* ncRNAs (Briese et al., 2016).

Post-transcriptional regulation of mRNAs in neurites by various classes of ncRNAs drive processes such as neurite outgrowth and synaptic plasticity. Such studies have tended to focus on regulatory RNAs, and thus these form the focus of discussion below.

### Short ncRNAs (sncRNAs)

Early studies investigating the subcellular distribution of sncRNAs in neurons focused on miRNAs. Canonically, transcribed primary miRNAs are processed into precursor miRNAs in the nucleus before being exported to the cytoplasm (O’Brien et al., 2018). Here, they form mature miRNAs around 22-nucleotides in length that can bind complementarily to mRNA targets to suppress their expression. Microarray studies have identified over 100 miRNAs in axons and growth cones, some enriched, and subsequent studies have revealed their importance in different aspects of axonal development and function (Natera-Naranjo et al., 2010; Han et al., 2011; Dajas-Bailador et al., 2012; Kaplan et al., 2013; Sasaki et al., 2014; Zhang et al., 2015). Microarray and RT-qPCR studies have also identified many miRNAs and their precursors, pre-miRNAs, in dendrites and synapses, along with Dicer and other proteins involved in miRNA biogenesis (Lugli et al., 2008, 2012). Enrichment of such precursors in synaptic fractions suggests additional compartmentalisation of local processing into functional miRNAs (Lugli et al., 2005, 2008).

Pre-miRNAs were found to associate with CD63-labelled vesicles, thought to represent late endosomes, for transport into axons (Vargas et al., 2016; Corradi et al., 2020). Intriguingly, the RNA-induced silencing complex (RISC), which is needed for miRNA processing, has also been shown to localise to axonal branch points and growth cones, a process that is facilitated by mitochondria (Gershoni-Emek et al., 2018). However, it is not clear whether the RISC functions directly on or adjacent to the vesicle to process co-trafficked pre-miRNAs, or whether it acts on different pre-miRNAs that already reside in the axon. Nevertheless, the presence of pre-miRNAs in distal regions of neurons suggests that these RNA precursors are processed locally to exert their function in response to environmental stimuli. Indeed, evidence has shown that pre-miRNAs are processed in axons and dendrites in response to injury (Kim et al., 2015) or neuronal excitation (Sambandan et al., 2017), respectively.

Recently, unbiased total RNA-Seq approaches have assayed the range of small ncRNAs in axons, dendrites, and synapses more globally. RNA-Seq performed following mouse embryonic spinal cord compartmentalized culture identified 401 miRNAs, with 34 enriched in neurites (Rotem et al., 2017). Several of the neurite-localized miRNAs were up- or down-regulated in neurons containing mutations causing the neurodegenerative disease, Amyotrophic lateral sclerosis (ALS), suggesting that perturbations in miRNA regulation may play a central role in driving neurodegeneration.

In another study investigating sncRNAs in mouse cortical neuron axons following compartmentalized primary culture, identified tRNA-derived small RNAs (tsRNAs) as the most enriched class (Mesquita-Ribeiro et al., 2021). Derived from tRNA genes, tsRNAs are cleavage fragments of around 14-50-nucleotides. The functions of such axonal tsRNAs were not addressed in the study, but generally they are reported to bind specific RBPs and mRNAs, proposed to act as regulators of translation and degradation (Zong et al., 2021; Tian et al., 2022). The second most abundant group was rRNA, reflective of ribosome localisation to axons. miRNAs represented < 10% of the small ncRNAs in axons, with just over 35 miRNAs making up 80% of the miRNA reads. Fragment RNAs derived from snRNA genes, particularly U1 and U2, were also detected. Subsequent sncRNA-Seq on axoplasm from rat dorsal and ventral root nerves *in vivo* revealed rRNA and miRNA as dominant, with tsRNAs well-represented and snRNAs also identified (Mesquita-Ribeiro et al., 2021). The same miRNAs were the most abundant in both the mouse cortical axon and rat axoplasm datasets.

A study investigating the non-coding transcriptome in synaptosomes purified from mouse hippocampus, identified 65 miRNAs and 37 snoRNAs (Epple et al., 2021). Intersecting the list of miRNAs with mRNAs that localize to synapses, revealed 98% of the mRNAs would be targeted, suggesting a high degree of local regulation by miRNAs at synapses. Compartmentalized culture of hippocampal neurons also allowed for the isolation of synapses for sequencing. These samples contain more neurite tissue compared to the synaptosomes sample, but are less prone to contamination by RNAs from other neural cell types. 57 miRNAs were identified, 17 of which were conserved with those in synaptosomes. This conserved group regulate 80% of synaptic mRNAs. Many of the other 48 miRNAs specific to synaptosomes have been previously reported to be released by exosomes from astrocytes, suggesting this may be their source in that dataset.

**FIGURE 5:**
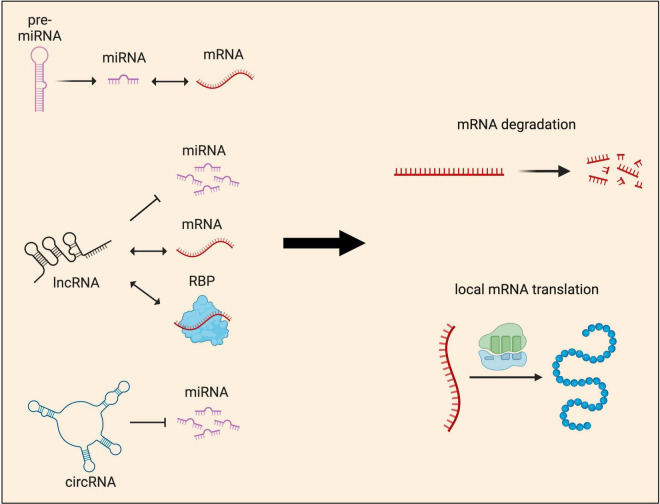
Local functions of non-coding RNAs in neurites. Non-coding RNAs (ncRNAs) influence gene expression at the post-transcriptional level by regulating either local mRNA translation and/or degradation. These two major outcomes are achieved through multiple mechanisms. miRNAs, which can be produced locally from pre-miRNAs, are known to interact directly with mRNAs to regulate their abundance and local protein synthesis. lncRNAs act as guides or scaffolds, interacting with both mRNAs and protein, but also compete with miRNAs to regulate local protein synthesis. circRNAs’ main mode of function is through acting as miRNA sponges, sequestering and preventing miRNAs from binding to their target mRNAs.

Thus far, functional studies of sncRNAs have mostly focused on miRNAs, which regulate mRNA targets by two mechanisms: translational repression and/or mRNA degradation (Baek et al., 2008; Bartel, 2009; Figure 5). Recent years have seen significant progress in our understanding of how miRNAs induce translational repression of local mRNAs. *miR-181d* was shown to mediate axon elongation in DRG neurons by repressing the local synthesis of MAP1B and CALM1 in response to NGF (Wang et al., 2015). Acting along similar lines, *miR-26a* and *miR-132* were shown to promote axon growth by repressing local protein synthesis of GSK3β and Rasa1, respectively (Hancock et al., 2014; Lucci et al., 2020). Moreover, *miR-181a* and *miR-182*, two highly abundant miRNAs in RGC axons, were shown to regulate the responsiveness of RGC axons to guidance cues by silencing the local translation of specific mRNA targets (Bellon et al., 2017; Corradi et al., 2020). Interestingly, recent work has also shown that upon exposure to axon guidance cues, pre-miRNAs are processed to miRNAs within RGC axons, silencing the basal translation of tubulin beta 3 class III (TUBB3) to enable accurate growth cone steering (Corradi et al., 2020). These findings support a model in which pre-miRNAs are stored within growth cones and synapses in an inactive form. Upon stimulation, rapid processing into active miRNAs for local translational repression ensures fast neuronal responses.

Together, these results provide experimental support for a model in which translational repression may be preferable over mRNA degradation in axons (Vo et al., 2010). Constitutive degradation of localized mRNAs that have been transported over long distances into axons would be inefficient or counterproductive. Moreover, while mRNA degradation is a terminal event, translational repression is reversible and can be employed for rapid response to internal or external cues.

In summary, functional studies of short ncRNAs have mostly focused on miRNAs thus far, which are particularly enriched in synaptic fractions and have the capability to target the entire local mRNA pool. Future studies could address miRNA and mRNA combinations occupying individual neurons to better understand the dynamics of such regulation. The functional impacts of tsRNAs, snoRNAs, and snRNAs in neurites and synapses is yet to be revealed and will likely form an important focus of future studies.

### Long ncRNAs (lncRNAs)

LncRNAs are generally defined as ncRNAs more than 200 nucleotides in length. They are enriched in the brain, where 40% of the tens of thousands that mammals possess are expressed (Briggs et al., 2015). Many are derived from protein-coding genes, being antisense, intronic, or intergenic in origin, while many others are pseudogenes (Mattick et al., 2023). LncRNAs are often spliced like mRNAs, and can be polyadenylated or not. *BC1/BC200* was the first lncRNA identified to localize to neurites, present in dendrites where it binds to various proteins and regulates local translation at synapses (Tiedge et al., 1991; Muslimov et al., 1997; Eom et al., 2011; Smalheiser, 2014; Briggs et al., 2015). *MALAT1* lncRNA also plays roles in synapse function, and both transcripts have been reported *in situ* in dendrites of mouse hippocampal pyramidal neurons (Alon et al., 2021).

Although many high-throughput sequencing datasets have globally characterized the transcriptomes of specifically neurites, most have focused on protein-coding transcripts. Typically, only handfuls of lncRNAs are highlighted, suggesting these datasets are untapped resources for identifying lncRNAs and aspects of mRNA regulation.

One study focusing on revealing lncRNAs more extensively in the rat spinal cord, though not at subcellular resolution, identified 772 transcripts differentially regulated following contusive injury, the majority (68%) upregulated (Zhou et al., 2018). This suggests that lncRNA functions are implicated in pathogenesis and limited repair capacity associated with spinal cord damage. Numerous specific neurite-localized lncRNAs have now been identified in various RNA-Seq datasets. In mouse embryonic motor axons, this includes the well reported on *MALAT1*, as well as *XIST, MIAT, RMST*, and *7SL* RNA, a component of the signal recognition particle, important for ER localisation of proteins (Briese et al., 2016). High-throughput sequencing of rat DRG neurons identified 3103 lncRNAs, the 20 most abundant of which were subsequently investigated for axonal enrichment (Wei et al., 2021). *ALAE* was shown to be the top candidate, important in axon growth through the regulation of *Gap43* local translation.

Studies focused on characterizing synaptic transcriptomes have typically covered lncRNAs in more detail. In one study, 6 high-confidence lncRNAs were identified in synaptosomes purified from mouse hippocampus (Epple et al., 2021). Strikingly, sequencing of synapses following a compartmentalized culture protocol where the tissue isolated includes more neurite material, identified 199 lncRNAs. This expanded group are associated with regulating oxidative phosphorylation and synaptic plasticity. Thus, this data suggests a wider range of lncRNAs localize to neurites than synapses than is currently understood. Another study characterizing lncRNAs from synaptoneurosomes of activated hippocampal neurons identified *Gm38257/ADEPTR* as the most enriched transcript compared to whole hippocampal neurons (Grinman et al., 2021). Derived from intron-1 of *Arl5b*, *Gm38257/ADEPTR* lncRNA is upregulated and trafficked to synapses upon activation, independent of Arl5b mRNA. The transcript acts as a scaffold, binding to ANKB and SPTN1 proteins for their transport to dendrites, and such transport is KIF2A-dependent.

Natural antisense transcripts are lncRNAs important for neurite development (Modarresi et al., 2012), and have been detected sitting alongside their complementary protein-coding sense transcripts in synaptoneurosomes isolated from adult mouse forebrain (Smalheiser et al., 2008). In some cases, the two transcripts are expressed at similar levels, while others exhibited significant differences in expression. The degree of interaction between these complementary transcripts in synaptoneurosomes is unclear.

Functionally, lncRNAs can act via several mechanisms to influence gene expression at the post-transcriptional level (Figure 5), and while their expression levels are often relatively low, they can exert great influence (Wu et al., 2021). They are increasingly found to be associated with RNA granules in axons and dendrites, indicating they may provide key functions to such membrane-less organelles. For instance, it is known that RNA granules with distinct RNPs can contribute to translational repression (Vessey et al., 2006). LncRNAs may associate with RNPs to form these granules (Khong et al., 2017) as *BC1* is known to associate with poly(A) binding protein (PABP), translation initiation factors and components of the ribosome at the synapse (Tiedge et al., 1991; Muddashetty et al., 2002; Lin et al., 2008). Indeed, RNA granules have been shown to play a role in synaptic plasticity and long-term memory formation (Solomon et al., 2007; Nakayama et al., 2017) by silencing translation and promoting RNA stability (Hubstenberger et al., 2017; Khong et al., 2017). Alternatively, lncRNAs within RNA granules can also rapidly facilitate local protein synthesis when translation is in high demand (Mazroui et al., 2007; Baez et al., 2011). Indeed, it was recently shown that an m^6^A-modified lncRNA *Dubr* binds YTHDF1/3 complex through its m^6^A modification, thereby preventing YTHDF1/3 complex from degradation via the proteasome pathway, facilitating translation of *Tau* and *Calmodulin*. Although it is not yet known whether *Dubr* acts in the cytoplasm or axons, this process was found to be essential for DRG axon elongation (Huang et al., 2022).

In distal parts of neurons, lncRNAs have been shown to work as guides or scaffolds. For example, *BC1* mediates translation silencing at the synapse by bridging the repressor FMRP and its target mRNAs (Zalfa et al., 2005; Lacoux et al., 2012; Briz et al., 2017). At the synapse, *BC1* can also bind to translation initiation factor, eIF4A, and PABP, preventing their interaction with target mRNAs to initiate translation (Muddashetty et al., 2002; Lin et al., 2008). Acting along similar lines, the lncRNA *NORAD* has been hypothesized to act as a decoy for dendrite-localized PUMILIO to prevent it from repressing translation (Vessey et al., 2010; Lee S. et al., 2016). Finally, the lncRNA *Meg3* was found to regulate AMPA receptor insertion to the plasma membrane, a process that has been hypothesized to be partly due to *Meg3* competition with miRNAs regulating PTEN/PI3K/AKT signaling pathway during synaptic plasticity in neurons (Tan et al., 2017). Despite these interesting lines of evidence, the functional relevance of lncRNAs in neurites and at the synapse is not fully understood, and future studies will likely provide new insight into the role of such localized lncRNAs.

### Circular RNAs (circRNAs)

CircRNAs are a highly stable class of RNAs formed from non-canonical back-splicing, where a downstream/3′ splice donor fuses with an upstream/5′ splice acceptor (Kristensen et al., 2022). They can contain exonic sequences only or include introns too. Also, intron lariats resulting from canonical pre-mRNA splicing can remain present as circRNAs if they evade linearisation by debranching enzymes (Kristensen et al., 2022). Both canonical splicing and back-splicing depend upon the spliceosome, and often, the two types of reaction are in competition on pre-mRNAs.

Investigations of circRNAs across various mouse tissues revealed their enrichment in the brain, and formation associated with neuronal differentiation (Rybak-Wolf et al., 2015; You et al., 2015; Dong et al., 2023). Such findings were observed across mammalian species. Comparing circRNAs in mouse and human brain samples, identified 15,849 and 65,731, respectively−the discrepancy likely in part due to deeper sequencing of human samples (Rybak-Wolf et al., 2015). Strikingly, 2,338 of the genes giving rise to circRNAs produce 10 or more circularized isoforms, which are frequently expressed at higher levels than linear mRNA counterparts (Rybak-Wolf et al., 2015; You et al., 2015).

CircRNAs are derived particularly from genes encoding synaptic proteins (Rybak-Wolf et al., 2015; You et al., 2015; Watts et al., 2023). Indeed, comparing expression between cell soma and neuropil in mouse, revealed that circRNAs are often enriched in neuropil more than linear mRNAs from the same genes. Similar results were also observed in rat samples, and a 23.6% overlap in the circRNAs in neuropil of the two species was observed (You et al., 2015; Saini et al., 2019). Furthermore, circRNAs were shown to be especially enriched in synaptosomes (Rybak-Wolf et al., 2015; You et al., 2015). Shifts in circRNA expression have been reported to occur with synaptogenesis, independent of overall host gene expression (You et al., 2015). Their levels can also be modulated by changes in neuronal activity and plasticity (You et al., 2015). CircRNAs derived from synaptic genes bind and are regulated by the neuronal-enriched splicing factor, SFPQ (Watts et al., 2023). The nature of such regulation is unclear, including where in the neuron it occurs given that in addition to its nuclear expession, SFPQ was recently reported to also localise to axons and dendrites (Cosker et al., 2016; Thomas-Jinu et al., 2017; Watts et al., 2023).

Functionally, ribosomal profiling data supports the consensus that while circRNAs may have roles in regulating local translation (Figure 5), they themselves are not translated (You et al., 2015). A circRNA from the gene encoding the nuclear lncRNA, *Rmst*, was highly enriched in dendrites and synapses, suggesting very distinct non-coding roles for circRNAs than the non-coding roles of linear isoforms (Rybak-Wolf et al., 2015). It has been demonstrated that circRNAs can functionally act as miRNA sponges, sequestering and preventing them from binding to their target mRNAs (Hansen et al., 2013; Memczak et al., 2013). For instance, *ciRS-7*, also known as *circCdr1as*, has more than 70 putative binding sites for the dendritically enriched *miR-7*, allowing multiple interactions (Hansen et al., 2013). Knockout of *ciRS-7* downregulated *miR-7* expression, whereas knockdown of *ciRS-7* decreased the expression of *miR-7* target genes (Hansen et al., 2013; Memczak et al., 2013; Piwecka et al., 2017). Although the specific function of circRNAs in neurites has not yet been addressed, these ncRNAs could similarly participate in the regulation of local protein synthesis.

## Neuron-to-neuron RNA transfer

Exosomes are small secretory extracellular vesicles (EVs) that play a role in intercellular communication by transporting a collection of biomolecules, including proteins, nucleic acids and lipids, between adjacent cells or over longer distances. RNAs in exosomes include mRNAs and ncRNAs like miRNAs (Valadi et al., 2007; Crescitelli et al., 2013; Xia et al., 2019). A recent investigation of sncRNAs in mouse primary cortical neurons identified exosomes were dramatically enriched for tsRNAs, with rRNAs also highly abundant, while miRNAs represent < 10% of their contents (Mesquita-Ribeiro et al., 2021). snoRNA-derived fragments were also present. The identification of coding and non-coding RNAs in exosomes (Figure 1) suggests such vesicles have the potential to influence the functional and molecular characteristics of recipient cells.

How RNAs are sorted into exosomes is not well understood. Some evidence for a passive sorting mechanism of RNAs into exosomes exists, however, recent literature has demonstrated that soluble RBPs could serve as key players, forming complexes with RNAs and transporting them into extracellular vesicles during the biosynthesis of exosomes (Villarroya-Beltri et al., 2013; McKenzie et al., 2016; Santangelo et al., 2016; Statello et al., 2018). Neuronal exosomes can also package mRNAs in association with proteins, such as the activity-regulated cytoskeleton-associated protein (ARC). As a master regulator of synaptic plasticity, ARC protein in exosomes encapsulates its own mRNA or other highly abundant mRNAs and traffics them between cells (Ashley et al., 2018).

The transfer of exosomes at synapses has long been proposed as a potential mechanism of cell-to-cell communication within the nervous system (Smalheiser, 2007). Studies on both developing and mature neurons have suggested that glutamatergic stimulation can induce exosome release (Fauré et al., 2006; Lachenal et al., 2011), demonstrating the involvement of synaptic activities in the process. Mounting evidence has revealed exosomes are a key modulator of synaptic activity under physiological conditions, as they contain neurite-associated miRNAs and mRNAs that modulate circuit formation and synaptic function after being internalized by local neurons (Morel et al., 2013; Goldie et al., 2014). For example, during circuit formation, BDNF mediates the sorting of specific miRNAs in neuron-derived exosomes (Antoniou et al., 2023). BDNF-induced exosomes in turn increase excitatory synapse formation in recipient hippocampal neurons, a mechanism dependent on inter-neuronal delivery of miRNAs (Antoniou et al., 2023). Depolarisation of differentiated human SH-SY5Y neuroblastoma cells was shown to be associated with an increase in exosomes enriched with primate specific miRNAs, whose mRNA targets are related to synaptic function (Goldie et al., 2014). These observations point to a mechanism where miRNA transfer across the synaptic cleft could influence local mRNA translation and degradation. Finally, blocking the trafficking of exosomes containing activity-regulated cytoskeleton-associated (*Arc*) mRNA from pre-synaptic terminals to post-synaptic muscle has been shown to result in dysregulation of synapse maturation and activity-dependent plasticity (Ashley et al., 2018).

## Future perspectives: revealing the scope of local splice isoform diversity using third-generation sequencing technologies

Huge strides have been made in understanding the genes whose RNAs (often of multiple RNA types) reside in axons, dendrites, and at synapses. However, relatively little is known regarding splice isoform diversity at such subcellular resolution. This is largely due to the nature of next-generation RNA sequencing technologies that have been the gold standard thus far, relying on short reads typically covering a single exon or single exon-exon junction. These datasets enable robust comparison of gene expression values across samples, and enable individual alternative splicing events comparison. However, analyses of the same dataset using different bioinformatic tools, has been reported to identify little overlap in splicing events identified, owing to varying requirements in mapped read distribution to detect events, emphasizing the need for new approaches (David et al., 2022). Furthermore, short-read splicing analyses are insufficient for providing insight regarding full-length splice isoform diversity. This requires a sequencing approach where RNA is not fragmented prior to reverse transcription, and hence does not utilize short reads.

Recent advancement in the development of third-generation sequencing technologies, producing long reads, are paving the way to revolutionize our understanding in this area. Identification of full-length transcriptomes with depth and breadth can now be achieved, with two techniques dominating. PacBio Iso-Seq involves sequencing cDNA following 3′ poly(A) tail primed reverse transcription, while Oxford Nanopore Technologies (ONT) sequencing can occur directly from RNA. Each technique offers its own advantages. PacBio Iso-Seq achieves > 99% accuracy, as each cDNA is sequenced many times to produce consensus HiFi reads (Wenger et al., 2019). ONT can sequence RNAs without poly(A) tails and can detect RNA modifications (e.g., methylation), as well as infer RNA structure (Wang et al., 2021). Both technologies are effective for sequencing transcripts < 10 kb in length, however, for especially long transcripts, reads are better detected by ONT sequencing, likely due to limitations in reverse transcription during PacBio sequencing library prep (Udaondo et al., 2021).

Transcripts from 95% human genes are prone to alternative splicing (Pan et al., 2008; Wang et al., 2008), and the process is particularly elaborate in the nervous system (Yeo et al., 2004; Barbosa-Morais et al., 2012; Raj and Blencowe, 2015). So far, long-read transcriptomic sequencing has been applied to developing and adult cortices in mouse and human (Leung et al., 2021; Patowary et al., 2023), revealing huge swathes of transcript isoforms that were not characterized by short-read RNA-Seq analyses. Given the broad nature of the samples used in these studies (sub-regions of cortical tissue), and limited depth of sequencing, it is highly likely that many more transcript isoforms remain uncovered.

PacBio long-read RNA sequencing has also been harnessed to reveal more accurately the extent of mRNA diversity for 30 genes encoding CNS cell-surface molecules in the mouse retina and brain (Ray et al., 2020). Some of the genes were known to generate many isoforms, but their full repertoires were not well characterized. The study identified hundreds of isoforms for some molecules, with *Nrxn3* showing over 750. In some cases, novel transcript isoforms showed far greater abundance than the canonical isoform. Expectedly, a higher number of transcript isoforms correlated with more protein isoforms, however, open reading frame (ORF) prediction identified that genes often have many more transcript isoforms than the number of ORFs, potentially indicating the presence of many uncharacterised lncRNAs. Inputting assembled transcripts from such datasets into tools such as CPAT (Wang et al., 2013), CPC2 (Kang et al., 2017) and Pfam (El-Gebali et al., 2019), may be used to determine the coding potential of transcripts on a greater scale. While the study examined splice isoform diversity in detail amongst this small subset of 30 genes, diversity amongst other classes of genes whose mRNAs are expressed locally such as those encoding ribosomal and mitochondrial proteins, remains largely uncovered.

Housekeeping ncRNAs reflect a huge amount of the total RNA in cells, with around 80% being rRNA and up to 15% tRNA (Deng et al., 2022). Indeed, local translation points towards an abundance of rRNA and tRNAs in axons, dendrites, and synapses, however, the specific proportions of each type of RNA within these subcellular compartments is largely unknown. Axonal ribosomes have been suggested to exhibit heterogeneity and undergo local remodeling (Shigeoka et al., 2019; Fusco et al., 2021). It is possible that housekeeping ncRNAs, including rRNAs, may also exhibit layers of cell type functional specificity (Ferretti and Karbstein, 2019). Although rRNA is not thought to undergo exchange in ribosomes (Mathis et al., 2017), with pre-rRNAs restricted to the nucleolus (Shigeoka et al., 2019), cell type- or even subcellular-specific differences in rRNAs could be exhibited in other ways (Ferretti and Karbstein, 2019). Changes in rRNA distribution, and chemical modifications affecting their stability or interaction with specific ribosomal proteins remain to be addressed by future studies.

Regarding regulatory ncRNAs, circRNAs pose a particularly intriguing, diverse class of underexplored highly abundant RNAs in neurites, with isoforms often more enriched in the periphery than linearised coding isoforms and understanding of their functions limited (Rybak-Wolf et al., 2015; You et al., 2015). The full extent of their diversity can be elucidated by long-read sequencing (Rahimi et al., 2021).

In conclusion, third-generation sequencing holds the power to provide significant advances towards revealing the true range of full-length mRNA and ncRNA splice isoforms present within far-flung neuronal subcellular compartments. This will enable the identification of alternative isoforms specific to axons versus dendrites versus synapses at new resolution. Single-cell based long-read sequencing will provide true insight into cell-specific isoform diversity. Altogether, such information will likely transform our understanding of the variety of ways by which individual genes are able to regulate their own expression, and that of other genes, to assert regulatory influence on local transcriptomes and proteomes.
